# Association of prediabetes with clinical outcomes in patients with chronic coronary syndrome: a post hoc analysis of the ISCHEMIA and ISCHEMIA-CKD trials

**DOI:** 10.1186/s12933-024-02232-z

**Published:** 2024-05-20

**Authors:** Anselm Jorda, Christian Hengstenberg, Irene M. Lang, Alexandra Kautzky-Willer, Jürgen Harreiter, Markus Zeitlinger, Bernd Jilma, Georg Gelbenegger

**Affiliations:** 1 Department of Clinical Pharmacology, Medical University of Vienna, Vienna, Austria; 2 Division of Cardiology, Department of Medicine II, Medical University of Vienna, Vienna, Austria; 3 Division of Endocrinology and Metabolism, Department of Medicine III, Medical University of Vienna, Vienna, Austria; 4 Department of Medicine, Landesklinikum Scheibbs, Scheibbs, Austria

**Keywords:** Impaired glucose tolerance, Stable coronary artery disease, Mortality, Prediabetes, Cardiovascular risk

## Abstract

**Background:**

There is conflicting evidence whether prediabetes is associated with adverse clinical outcomes in patients with chronic coronary syndrome. We aimed to assess the effect of prediabetes in patients with chronic coronary syndrome on clinical outcomes.

**Methods:**

This is a secondary analysis of data from the ISCHEMIA and ISCHEMIA-CKD trials, including patients with chronic coronary syndrome determined by coronary computed tomography angiography or exercise-stress testing. Participants were assigned to the normoglycemia group (HbA1c < 5.7% [< 39 mmol/mol]), prediabetes group (HbA1c 5.7–6.4% [40–47 mmol/mol]), or diabetes group (HbA1c ≥ 6.5% [≥ 48 mmol/mol]). The primary end point of this study was all-cause mortality. Secondary endpoints included major adverse cardiovascular events and composites thereof.

**Results:**

Overall, the primary endpoint all-cause mortality occurred in 330 (8.4%) of 3910 patients over a median follow-up time of 3.1 years (IQR 2.1–4.1). The primary endpoint all-cause mortality occurred in 37 (5.2%) of 716 patients in the normoglycemia group, in 63 (6.9%) of 911 in the prediabetes group, and in 230 (10.1%) of 2283 in the diabetes group. In the covariate-adjusted Cox model analysis, the estimated adjusted HR (aHR) in the prediabetes group as compared with the normoglycemia group was 1.45 (95%CI, 0.95–2.20). The aHR in the diabetes group as compared with the normoglycemia group was 1.84 (95%CI, 1.29–2.65). Prediabetes, compared with normoglycemia, was associated with an increased risk of stroke (aHR, 3.44, 95%CI, 1.15–10.25). Subgroup analyses suggested an increased risk of all-cause death associated with prediabetes in males and patients under 65 years.

**Conclusions:**

In patients with chronic coronary syndrome, diabetes but not prediabetes was associated with significantly increased risk of all-cause death within a median follow-up period of 3.1 years.

Trial Registration NCT01471522, BioLINCC ID 13936.

**Supplementary Information:**

The online version contains supplementary material available at 10.1186/s12933-024-02232-z.

## Introduction

Diabetes mellitus is a rapidly progressing epidemic health issue strongly associated with coronary artery disease (CAD) [1–3]. In CAD, diabetes is associated with worse clinical outcomes [4, 5] and therefore requires rigorous treatment [6]. Prediabetes is a state of impaired carbohydrate metabolism that does not meet the diagnostic criteria of diabetes but is associated with a high risk of progression into it [7]. According to the current 2023 American Diabetes Association (ADA) and 2023 European Society of Cardiology (ESC) guidelines, prediabetes is defined as a fasting plasma glucose of 5.6–6.9 mmol/L (100–125 mg/dL), glycated hemoglobin (HbA1c) of 39–47 mmol/mol (5.7–6.4%), or a 2-h oral glucose tolerance test glucose of 7.8–11.0 mmol/L (140–199 mg/dL) [6]. Similar to patients with diabetes, patients with prediabetes show an increased risk of developing cardiovascular disease, in particular CAD [8, 9]. However, there are conflicting data available about the prognostic value of prediabetes in patients with established chronic coronary syndrome. Data from a recently published meta-analysis, pooling studies of patients with atherosclerotic cardiovascular disease, found an increased risk of all-cause mortality in patients with atherosclerotic cardiovascular disease and prediabetes [8]. The ISCHEMIA and ISCHEMIA-CKD trials enrolled and randomized patients with stable coronary artery disease (now “chronic coronary syndrome”) to an initial invasive strategy (coronary angiography and—if feasible—revascularization with optimal medical therapy) or to an initial conservative strategy (optimal medical therapy alone) [10, 11].

Impaired glucose metabolism is highly prevalent in patients with chronic coronary syndrome, with diabetes affecting almost 50% of patients in the ISCHEMIA trials. Given that a substantial proportion of non-diabetic patients fulfill the diagnostic criteria of prediabetes, we aimed to assess the association between prediabetes and clinical outcomes in patients from the ISCHEMIA and ISCHEMIA-CKD trials.

## Methods

This secondary analysis pooled data from the ISCHEMIA and the ISCHEMIA-CKD trials and was performed according to the Strengthening the Reporting of Observational Studies in Epidemiology (STROBE) recommendations [10, 11]. Data was obtained from the NHLBI Biologic Specimen and Data Repository Information Coordinating Center [12]. The exact design and primary results of the ISCHEMIA trials have been reported [13, 14]. In brief, the ISCHEMIA trial included patients with chronic coronary syndrome and moderate-to-severe ischemia on clinically indicated stress imaging or severe ischemia on exercise testing. Patients were eligible if they were clinically stable, including stable angina or silent ischemia. The ISCHEMIA-CKD trial included patients who additionally had advanced chronic kidney disease. Thus, patients with an eGFR above 30 ml/min/1.73m^2^ were included in the ISCHEMIA trial; patients with an eGFR below 30 ml/min/1.73m^2^ were included in the ISCHEMIA-CKD trial. Most patients with normal kidney function (eGFR ≥ 60 ml/min/1.73m^2^) underwent coronary computed tomography angiography with the intent to rule out ≥ 50% left main stenosis or < 50% stenosis of any epicardial artery. Coronary computed tomography angiography was not performed in patients with an eGFR < 60 mL ml/min/1.73m^2^ to avoid acute kidney injury. Key exclusion criteria were a recent acute coronary syndrome, unprotected left main stenosis ≥ 50%, a left ventricular ejection fraction ≤ 35%, NYHA class III or IV heart failure and unacceptable angina. In both trials, patients were randomly assigned either to be managed with an initial invasive strategy consisting of coronary angiography and—if appropriate—revascularization added to medical therapy or an initial conservative strategy consisting of medical therapy alone. Both trials did not find evidence that an initial invasive strategy, as compared with an initial conservative strategy, reduced the risk of the respective primary outcomes. The trials were approved by the NYU School of Medicine Institutional Review Board.

### Trial population and group definitions

This secondary analysis included subjects from both trials. Because the two treatment strategies (initial invasive or conservative strategy) yielded similar clinical outcomes, patients were pooled irrespective of their group assignment. In addition, pooling the two treatment groups may better reflect the real-world setting in which both strategies are currently being employed. The interaction between glycemic state and treatment effect was not evaluated. A previous analysis did not show a benefit for invasive management in patients with or without diabetes [5]. To evaluate the impact of prediabetes on clinical outcomes, subjects were then classified into three groups based on their baseline HbA1c (%): (i) normoglycemia (HbA1c < 5.7%, < 39 mmol/mol), (ii) prediabetes (HbA1c 5.7–6.4%, 40–47 mmol/mol), or (iii) diabetes (HbA1c ≥ 6.5%, ≥ 48 mmol/mol). Participants without available HbA1c at baseline were excluded. To assess the impact of different definitions of prediabetes on clinical outcomes, we performed the primary and secondary analyses based on baseline fasting glucose level instead of baseline HbA1c levels. Group assignment according to fasting glucose was performed following established definitions (normoglycemia: ≤ 5.5 mmol/L or ≤ 99 mg/dL, prediabetes: 5.6–6.9 mmol/L or 100-125 mg/dL and diabetes: ≥ 7.0 mmol/L or ≥ 126 mg/dL) [7]. Subjects receiving glucose-lowering medication including insulin were assigned to the diabetes group, irrespective of their baseline HbA1c. Of note, sodium glucose transporter 2 inhibitors were not approved for use in non-diabetic patients with heart failure or chronic kidney disease at the time of enrollment and therefore did not result in erroneous assignment of non-diabetic patients to the diabetes group.

### Outcomes

The primary end point of this study was all-cause mortality. Secondary endpoints were the primary composite endpoint from the ISCHEMIA-CKD trial (all-cause mortality or myocardial infarction); the primary composite endpoint of the main ISCHEMIA trial (cardiovascular death, myocardial infarction, or hospitalization for unstable angina, heart failure, or resuscitated cardiac arrest); the composite endpoint of cardiovascular death, myocardial infarction, or stroke; the individual components (cardiovascular death, myocardial infarction, heart failure, and stroke); and the initiation of new dialysis. Endpoint definitions have previously been published. [10] In an exploratory analysis, we examined the interaction between glycemic status (normoglycemia, prediabetes or diabetes) and the treatment effect (invasive versus conservative strategy) of the ISCHEMIA trials.

### Statistical analyses

Categorical and continuously measured patient baseline characteristics were summarized using numbers with percentage (%) or medians with interquartile ranges (IQR), respectively. Baseline group differences were tested using the Fisher exact test and Kruskal–Wallis test as appropriate. Outcomes were analyzed in time-to-event analyses using the Kaplan–Meier method. The prediabetes and diabetes groups were compared with the normoglycemia group using a Cox proportional-hazards model to estimate the average risk increase according to the glycemic status. P values of the time-to-event analyses have been calculated using the log-rank test. To assess the proportional hazards assumption, Schoenfeld residuals from the fitted models have been calculated. No violation of proportional hazards associations in Cox models was found for any of the outcomes. Results are reported as hazard ratios and 95% confidence intervals (CI). The confidence intervals of the secondary outcomes have not been adjusted for multiple comparisons. To account for differences between the three groups, the primary analysis was based on a Cox model adjusted for the covariates that represent the most important risk factors for the development of prediabetes and diabetes and the occurrence of adverse cardiovascular events: sex, age at randomization, estimated glomerular filtration rate, hypertension, smoking status, low-density lipoprotein (LDL) levels at baseline and BMI [15]. Multiple subgroup analyses have been performed to assess the heterogeneity of the risk associated with prediabetes and diabetes. As exploratory analyses, the cumulative incidence of diabetes in the normoglycemia and prediabetes group was assessed. Moreover, all-cause mortality was compared between patients with prediabetes who did or did not develop diabetes, according to an HbA1c of 6.5% or higher at follow-up visits. The sample size calculation of this secondary analysis assumed that patients without diabetes were equally divided into patients with normoglycemia and prediabetes. Based on a two-sided alpha of 0.05 and 80% power to detect a HR of 1.5, considering a mortality rate of 9%, our sample size calculation indicated that a total of 1600 patients (i.e., the sum of patients with normoglycemia and prediabetes) would be needed to detect a statically significant difference using the Cox proportional hazard model. All analyses were conducted in R statistical software.

## Results

### Study participants

**Fig. 1:**
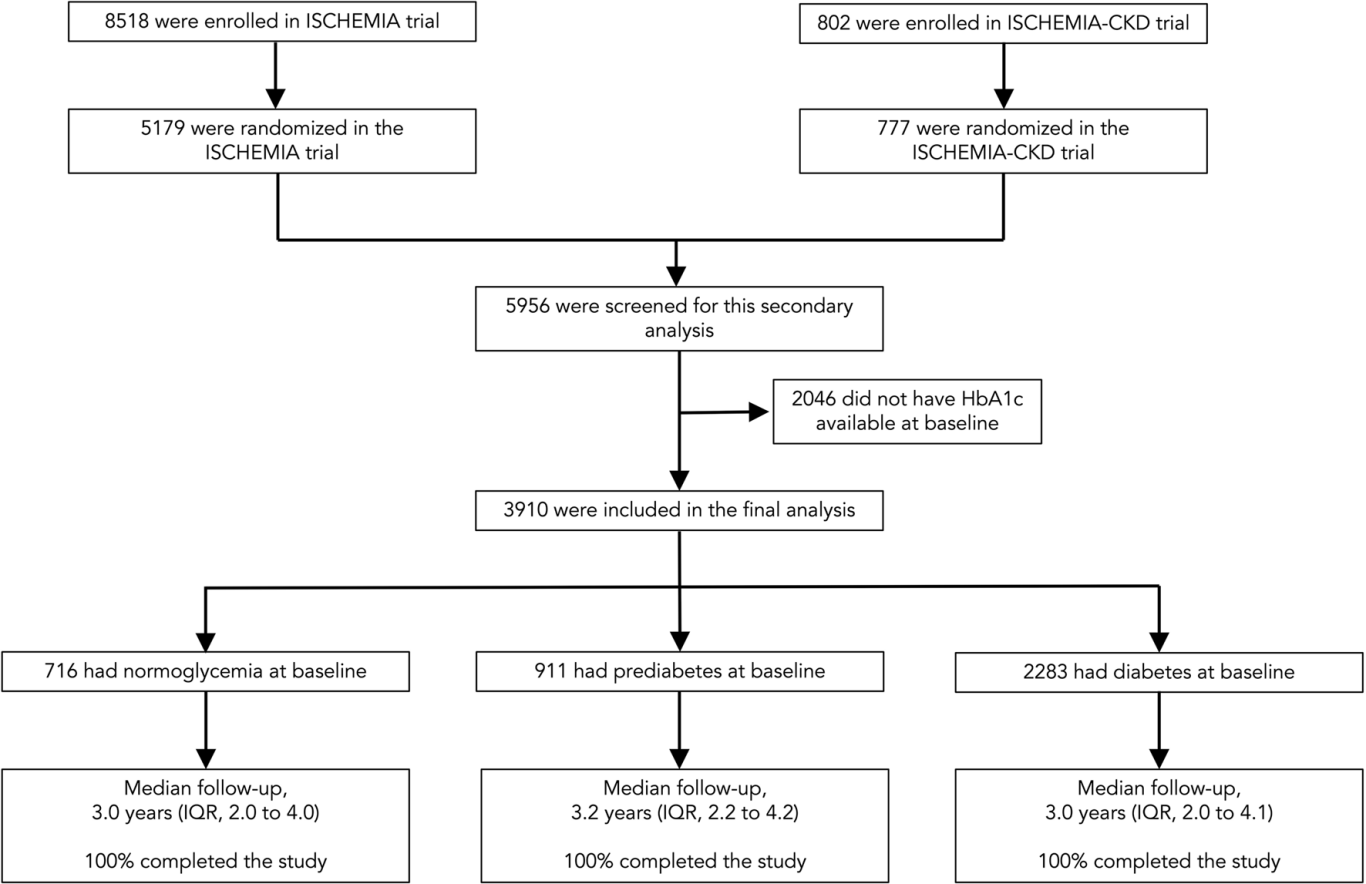
Study flow chart

Figure 1 provides the flow diagram of this study. Participants randomized in the ISCHEMIA trial (n = 5179) and the ISCHEMIA-CKD trial (n = 777) were screened for eligibility for this analysis. Participants were enrolled from July 2012 through January 2018. After excluding 2046 patients without HbA1c levels available at baseline, we included 3910 patients in the final analysis. Of these 3910 patients, 716 (18.3%) had normoglycemia, 911 (23.3%) had prediabetes, and 2283 (58.4%) had diabetes. The overall median follow-up period was 3.1 years (IQR 2.1 to 4.1). The study was completed by 100% of the participants.

Baseline characteristics of the overall study population and the three groups are provided in Table 1. The overall median age was 64 years (IQR 58–70), without clinically relevant group differences. Only 893 (22.8%) of the 3910 participants were female. The normoglycemia group had fewer females than the prediabetes and diabetes groups (19.4% vs 21.8% vs 24.8%, respectively). Of the 2283 patients in the diabetes group, 715 (31.3%) required treatment with insulin. Overall, patients in the diabetes group had more comorbidities than patients in the normoglycemia and prediabetes groups, in which comorbidities were similar. Treatment assignment to invasive or conservative strategy was balanced between the three groups (Table 1).

**Table 1:** Baseline characteristics

	Overall	Normoglycemia	Prediabetes	Diabetes	p
n	3910	716	911	2283	
Median age (IQR)—yr	64 [58, 70]	64 [56, 70]	65 [58, 72]	64 [58, 70]	0.005
Female sex—no. (%)	893 (22.8)	139 (19.4)	199 (21.8)	555 (24.3)	0.018
Median body mass index (IQR)—kg/m^2^	27 (24, 31)	26 (23, 30)	27 (24, 30)	28 (25, 32)	< 0.001
Obesity (BMI > 30 kg/m^2^)—no. (%)	1367 (35.0)	185 (25.8)	263 (28.9)	919 (40.3)	< 0.001
Racial group—no./total no. (%)					< 0.001
Black or African American	222 (5.8)	32 (4.5)	47 (5.2)	143 (6.4)	
Other	1230 (31.9)	171 (24.1)	298 (33.1)	761 (33.8)	
White	2405 (62.4)	506 (71.4)	554 (61.6)	1345 (59.8)	
Ethnic group—no./total no. (%)					0.017
Hispanic or Latino	603 (15.4)	127 (17.7)	137 (15.0)	339 (14.8)	
Not Hispanic or Latino	3058 (78.2)	555 (77.5)	727 (79.8)	1776 (77.8)	
Unknown	249 (6.4)	34 (4.7)	47 (5.2)	168 (7.4)	
Median Hemoglobin A1c (IQR)—%	6.3 [5.7, 7.5]	5.4 [5.2, 5.6]	6.0 [5.8, 6.2]	7.2 [6.5, 8.3]	< 0.001
Use of insulin—no. (%)	715 (18.3)	0 (0.0)	0 (0.0)	715 (31.3)	< 0.001
Cigarette smoking—no./total no. (%)					0.001
Current Smoker	452 (11.6)	99 (13.8)	122 (13.4)	231 (10.1)	
Former Smoker	1760 (45.1)	340 (47.5)	411 (45.2)	1009 (44.2)	
Never Smoked	1694 (43.4)	277 (38.7)	376 (41.4)	1041 (45.6)	
Hypertension—no. (%)	2964 (75.8)	477 (66.6)	628 (68.9)	1859 (81.4)	< 0.001
Family history of premature coronary artery disease—no. (%)	872 (22.3)	171 (23.9)	201 (22.1)	500 (21.9)	0.889
Previous myocardial infarction—no./total no. (%)	730 (18.7)	111 (15.5)	171 (18.8)	448 (19.6)	0.159
Previous CABG—no./total no. (%)	150 (3.8)	13 (1.8)	31 (3.4)	106 (4.6)	0.002
Heart failure—no. (%)	199 (5.1)	30 (4.2)	22 (2.4)	147 (6.4)	< 0.001
Median ejection fraction (IQR)—%	60 [55, 65]	60 [55, 65]	60 [55, 65]	60 [54, 65]	0.026
History of atrial fibrillation or atrial flutter—no. (%)	169 (4.3)	39 (5.4)	34 (3.7)	96 (4.2)	0.222
History of cerebrovascular disease—no. (%)	141 (3.6)	23 (3.2)	19 (2.1)	99 (4.3)	0.007
History of peripheral‐artery disease—no. (%)	180 (4.6)	27 (3.8)	29 (3.2)	124 (5.4)	0.011
History of angina—no. (%)	3397 (86.9)	618 (86.4)	805 (88.4)	1974 (86.5)	0.328
Median SAQ Angina Frequency score (IQR)	90 [70, 100]	90 [70, 100]	90 [70, 100]	90 [70, 100]	0.518
Included in the CKD study—no. (%)	471 (12.0)	76 (10.6)	49 (5.4)	346 (15.2)	< 0.001
Median eGFR (IQR)—(mL/min/1.73m2)	79 [60, 95]	82 [65, 99]	82 [68, 96]	76 [55, 94]	< 0.001
Group assignment in main trial					
Invasive strategy	1960 (50.1)	354 (49.4)	474 (52.0)	1132 (49.6)	0.422
Conservative strategy	1950 (49.9)	362 (50.6)	437 (48.0)	1151 (50.4)	

#### Primary outcome

**Fig. 2:**
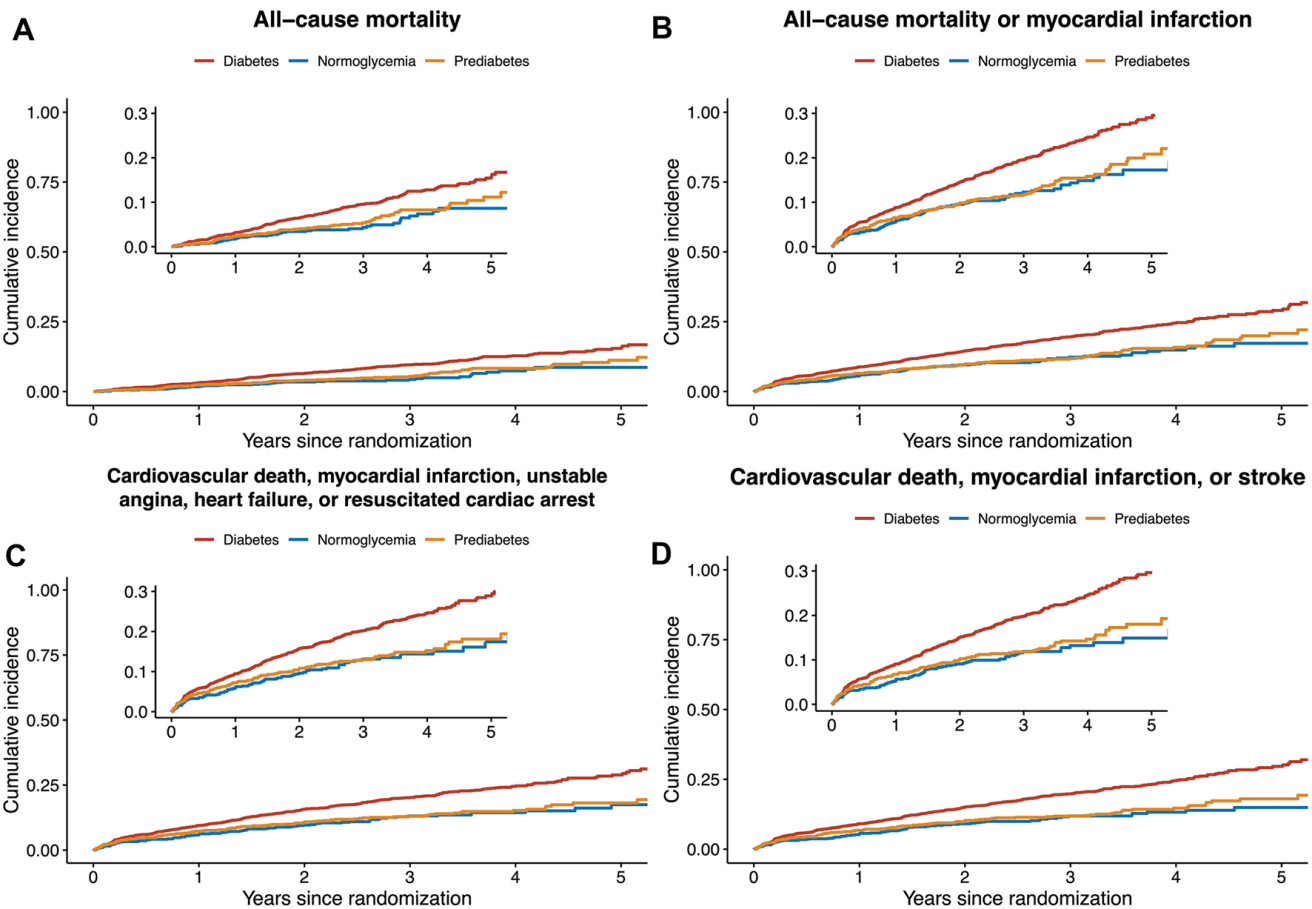
Time-to-event curves for the primary outcome and key secondary outcomes

Overall, the primary endpoint all-cause mortality occurred in 330 (8.4%) of 3910 patients (Table 2 and Fig. 2). The primary endpoint occurred in 37 (5.2%) of 716 patients in the normoglycemia group, in 63 (6.9%) of 911 in the prediabetes group, and in 230 (10.1%) of 2283 in the diabetes group. In the covariate-adjusted Cox model analysis, the estimated hazard ratio in the prediabetes group as compared with the normoglycemia group was 1.45 (95%CI, 0.95–2.20; P = 0.08). The estimated hazard ratio in the diabetes group as compared with the normoglycemia group was 1.84 (95%CI, 1.29–2.65; P = 0.001). The unadjusted Cox model analysis is provided in Table 2.

**Table 2:** Primary and secondary study outcomes

Study Endpoints	Normoglycemia (n = 716)	Prediabetes (n = 911)	Diabetes (n = 2283)	Pre-diabetes vs Normoglycemia		Diabetes vs Normoglycemia	
				unadjusted HR	adjusted HR	unadjusted HR	adjusted HR
All-cause mortality	37 (5.2)	63 (6.9)	230 (10.1)	1.27 (0.85–1.91)	1.45 (0.95–2.20)	1.93 (1.37–2.74)	1.84 (1.29–2.65)
All-cause mortality or myocardial infarction	73 (10.2)	104 (11.4)	389 (17.0)	1.10 (0.83–1.45)	1.11 (0.83–1.47)	1.63 (1.3–2.1)	1.48 (1.16–1.89)
Cardiovascular death, myocardial infarction, unstable angina, heart failure, or resuscitated cardiac arrest	84 (11.7)	12 (13.3)	433 (19.0)	1.09 (0.83–1.44)	1.09 (0.82–1.47)	1.65 (1.3–2.08)	1.43 (1.13–1.83)
Cardiovascular death, myocardial infarction, stroke	77 (10.8)	115 (12.6)	429 (18.8)	1.13 (0.85–1.51)	1.15 (0.86–1.55)	1.79 (1.40–2.28)	1.61 (1.26–2.08)
Cardiovascular death	25 (3.5)	44 (4.8)	186 (8.1)	1.32 (0.81–2.16)	1.54 (0.93–2.55)	2.32 (1.52–3.52)	2.14 (1.39–3.30)
Myocardial Infarction	56 (7.8)	74 (8.1)	260 (11.4)	1.00 (0.70–1.42)	0.95 (0.66–1.36)	1.48 (1.11–1.97)	1.31 (1.0–1.76)
Heart failure	12 (1.7)	14 (1.5)	57 (2.5)	0.86 (0.40–1.90)	0.74 (0.34–1.64)	1.49 (0.8–2.78)	0.95 (0.5–1.80)
Stroke	5 (0.7)	17 (1.9)	58 (2.5)	2.58 (0.95–7.0)	3.44 (1.15–10.25)	3.64 (1.46–9.06)	4.08 (1.47–11.3)
Initiation of new dialysis	5 (0.8)	8 (0.9)	54 (3.5)	1.16 (0.38–3.53)	1.01 (0.31–3.30)	3.37 (1.35–8.41)	1.34 (0.51–3.5)

#### Secondary outcomes

The composite endpoint of all-cause mortality or nonfatal myocardial infarction occurred in 73 (10.2%) of 716, 104 (11.4%) of 911, and 389 (17.0%) of 2283 patients in the normoglycemia, prediabetes and diabetes group, respectively (Table 2 and Fig. 2). There was a statistically significant difference between the diabetes and normoglycemia groups (adjusted HR, 1.48, 95%CI, 1.16–1.89), but not between the prediabetes and normoglycemia groups (adjusted HR, 1.09, 95%CI, 0.83–1.47). Compared with the normoglycemia group, the estimated risk of stroke was increased in the prediabetes group (adjusted HR, 3.44, 95%CI, 1.15–10.25) and in the diabetes group (adjusted HR, 4.08, 95%CI, 1.47–11.3). The risk of initiation of new dialysis was similar between patients in the prediabetes and the normoglycemia groups (adjusted HR, 1.01, 95%CI, 0.31–3.30). Compared with normoglycemia, diabetes was associated with a statistically significant increase in initiation of new dialysis before (HR, 3.37, 95%CI, 1.35–8.41) but not after adjustment for covariates (adjusted HR, 1.34, 95%CI, 0.51–3.50). The risk of the remaining secondary outcomes, except for heart failure, was higher in the diabetes group than in the normoglycemia group but comparable between the prediabetes and normoglycemia groups. (Table 2).

### Heterogeneity of prediabetes and diabetes effect

**Fig. 3:**
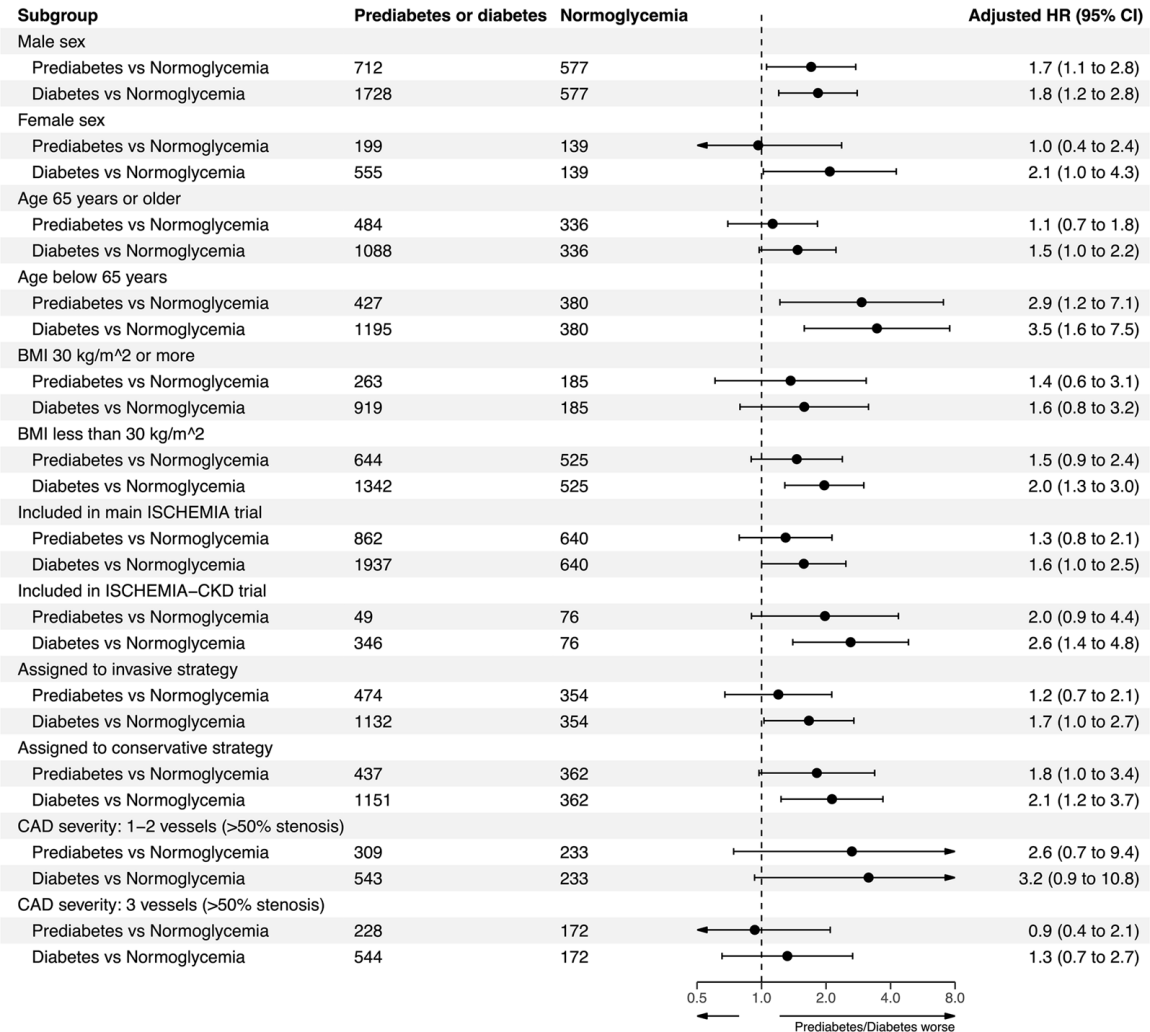
Subgroup analyses for the primary endpoint all-cause mortality (point estimates and confidence intervals are based on covariate adjusted Cox regression analyses)

Figure 3 depicts the estimated risks associated with prediabetes and diabetes across multiple subgroups. Unlike in female subjects (adjusted HR, 0.96, 95%CI, 0.39–2.36), prediabetes in male subjects was associated with an increased risk of all-cause death, as compared with the normoglycemia group (adjusted HR, 1.70, 95%CI, 1.06–2.75). Moreover, prediabetes was associated with higher mortality in patients below the age of 65 years (adjusted HR, 2.93, 95%CI, 1.22–7.06), but not in those aged 65 years or older (adjusted HR, 1.13, 95%CI, 0.70–1.82). Further evidence of heterogeneity was not found (Fig. 3).

### Exploratory analyses

There was no reduction in all-cause mortality of an invasive treatment strategy for stable coronary artery disease compared with a conservative strategy in patients with normoglycemia (adjusted HR 1.21, 95% CI 0.61–2.38), in patients with prediabetes (adjusted HR 0.88, 95% CI 0.53–1.45), and in patients with diabetes (adjusted HR 1.0, 95% CI 0.77–1.30).

The association between HbA1c values at baseline and the risk of all-cause death was plotted descriptively in Supplementary Fig. 1. Supplementary Fig. 2 shows the cumulative incidence of patients in the normoglycemia group and prediabetes group progressing to diabetes, according to follow-up HbA1c levels. At one year, 2.8% of patients in the normoglycemia group and 17.1% in the prediabetes group developed diabetes. At the end of the observation period (at 6 years) 6% in the normoglycemia group and 27.4% in the prediabetes groups developed diabetes. Progression from prediabetes to diabetes occurred in 45 (22.6%) of 199 females and in 205 (28.8%) of 712 males (OR 0.72 95%CI, 0.49–1.06). In the prediabetes group, there was no difference in all-cause mortality between patients developing diabetes (15 [6%] of 250) and patients not developing diabetes (48 [7.3%] of 661) within the observational period (adjusted HR, 0.69, 95%CI, 0.38–1.26) (Supplementary Fig. 3).

Fasting glucose was available in 549 patients with normoglycemia, 440 patients with prediabetes, and 2152 patients with diabetes. Group comparisons were similar between the primary analysis based on baseline HbA1c and the sensitivity analysis based on baseline fasting glucose levels. (Supplementary Table 1).

## Discussion

In this secondary analysis of the ISCHEMIA and ISCHEMIA-CKD trials comprising a total of 3910 patients with chronic coronary syndrome, all-cause mortality was similar between patients with prediabetes and patients with normoglycemia, with 95% confidence intervals of the adjusted hazard ratio ranging from 0.95 to 2.20. Similarly, there was no significant difference in cardiovascular death and in several composite endpoints of cardiovascular events in patients with normoglycemia and prediabetes. In contrast, all of these endpoints differed significantly between patients with normoglycemia and diabetes, which is in line with a large body of existing literature [5]. The lack of significant difference between normoglycemia and prediabetes contrasts the results from a recent meta-analysis that suggested prediabetes to be associated with an increased all-cause mortality in patients with established atherosclerotic cardiovascular disease [8]. Notably, meta-analyses of observational data are inherently susceptible to bias due to the potential for confounding variables, variability in study design, and the presence of publication bias, which collectively can distort the pooled estimates of effect. Nonetheless, an important strength of meta-analysis is the large sample size and statistical power, which are provided by the current study. In line with our observations, the ARTEMIS study found more adverse clinical outcomes in patients with coronary artery disease and diabetes but similar outcomes between patients with prediabetes and normoglycemia. [16] Similar to our study, the ARTEMIS study included a mostly low-to-intermediate risk population with coronary artery disease. A secondary analysis from the PROSPECT study yielded similar results in patients with acute coronary syndrome. [17]

Within one year, 17% of patients with prediabetes progressed to diabetes. Given the high prevalence of prediabetes worldwide and the substantial progression rate into diabetes and its associated risks, optimal management of patients with chronic coronary syndrome and prediabetes is of considerable importance [18, 19]. In our study, patients who progressed from prediabetes to diabetes had a similar cumulative risk of all-cause death as compared with patients who had no progression from prediabetes to diabetes. Because duration of diabetes has been shown to be a key prognostic factor of clinical outcomes, the limited observation period may explain the lack of observable difference in mortality in the initial years after diabetes onset [20, 21]. Consequently, prediabetes may constitute a phase in which only a limited amount of micro- and macrovascular damage has occurred, a hypothesis that may also be mirrored by a similar kidney function at baseline and initiation of new dialysis between patients with prediabetes and normoglycemia. Another study found no significant differences between patients with normoglycemia and prediabetes with regards to vulnerable coronary plaques [17], a finding that is supported by a similar incidence of nonfatal myocardial infarction between these two groups in our study.

In the overall analysis, stroke occurred more often in the prediabetes group than in the normoglycemia group. This finding affirms the results from a 2012 meta-analysis describing an increased risk of stroke in patients with prediabetes [22]. Notably, because secondary outcomes were not adjusted for multiple comparisons, this could be a false positive finding.

In two patient populations, males and patients aged under 65 years, prediabetes was associated with a higher risk of all-cause death compared with normoglycemia. Men are known to develop cardiovascular disease at an earlier age than women, and mortality from coronary heart disease also remains higher in men. [23] In addition, men are more likely to be diagnosed with diabetes at an earlier age [24]. Men were less likely to have adequate control of diabetes in a large US cohort [25]. Numerically more men than women with prediabetes developed diabetes in our study, without reaching statistical significance. While more data are needed to confirm this finding, our data could suggest that prediabetes is of greater clinical importance in men than in women, especially in those who are under 65 years. [26]

In the early 2000s, findings from the Diabetes Prevention Program Study showed that metformin reduces the onset of new diabetes in patients with prediabetes, which was believed to be of clinical importance [27]. A 20-year follow-up study, however, confirmed that the treatment of prediabetes with metformin did not translate into improved clinical outcomes, questioning its therapeutic use in prediabetes [28]. Consequently, current management of prediabetes is limited to preventative measures, such as lifestyle modification and reduction of risk factors to avoid disease progression [29]. Metformin is currently not recommended for the treatment of prediabetes. A substantial number of patients with prediabetes do not develop diabetes or even return to normal glucose regulation [30], which might render early pharmacologic treatment obsolete or even put patients at risk of drug-associated side effects.

In general, prediabetes alone is not an indication for antidiabetic drug therapy. Whether there are specific subgroups (e.g., men under the age of 65 years or patients with chronic kidney disease), who might potentially benefit from pharmacological treatment, can only be shown in large, randomized trials. Such trials would require an adequate follow-up period and sample size to detect differences in clinically relevant endpoints. Current evidence suggests that patients with prediabetes should be monitored more closely to detect progression to diabetes as soon as possible. This is further supported by the fact that there is often a considerable delay from onset of diabetes to diagnosis and treatment [31], at which point diabetic complications might have already occurred. [32–34]

### Limitations

This study has several limitations. Given this study is a post-hoc analysis of a prospective, randomized trial, our results should be considered hypothesis-generating. We could not report on the duration of prediabetes before enrolment in the study, which might influence the risk of death and clinical events. The ISCHEMIA trial did not differentiate between type 1 and 2 diabetes, however, a preponderance of type 2 diabetes (90–95%) is suggested [35, 36]. Importantly, patients with acute coronary syndrome, clinically significant left main coronary artery disease, and advanced heart failure were excluded from the ISCHEMIA and ISCHEMIA-CKD trials. The sample size of this study was too small to detect subtle group differences of the primary endpoint, subgroup differences, or endpoints with lower event rates. The confidence intervals of the effect estimate cannot exclude a clinically relevant survival difference between patients with normoglycemia and patients with prediabetes. Although the Cox regression model was adjusted for several covariates, residual confounding cannot be excluded. Finally, two thirds of included patients were of White race, resulting in underrepresentation of patients of Black race and Hispanic/Latino ethnicity. Results of this study may be interpreted cautiously for these patient populations.

## Conclusion

In patients with chronic coronary syndrome, diabetes but not prediabetes was associated with significantly increased risk of all-cause death within a median follow-up period of 3.1 years. Subgroup analyses showed that prediabetes was associated with a higher mortality in males and patients under the age of 65 years.

### Supplementary Information

Additional file1 (DOCX 234 kb)

## Data Availability

The data that support the findings of this study are available from the NHLBI Biologic Specimen and Data Repository Information Coordinating Center but restrictions apply to the availability of these data, which were used under license for the current study, and so are not publicly available.
