# Supplementary material for: Association of prediabetes with clinical outcomes in patients with chronic coronary syndrome: a post hoc analysis of the ISCHEMIA and ISCHEMIA-CKD trials
Source: Cardiovasc Diabetol. 2024 May 20;23:176. doi: 10.1186/s12933-024-02232-z (PMC11106853; doi:10.1186/s12933-024-02232-z)
Supplement: Supplementary file 1 — Additional file1 (DOCX 234 kb) [file 12933_2024_2232_MOESM1_ESM.docx]

# Supplementary Appendix

**Association of Prediabetes With Clinical Outcomes in Patients With Chronic Coronary Syndrome:**

A Post Hoc Analysis of the ISCHEMIA and ISCHEMIA-CKD Trials

Anselm Jorda^1^, Christian Hengstenberg^2^, Irene M. Lang^2^, Alexandra Kautzky-Willer^3^, Jürgen Harreiter^3,4^, Markus Zeitlinger^1^, Bernd Jilma^1^, Georg Gelbenegger^1^*

^1^Department of Clinical Pharmacology, Medical University of Vienna, Vienna, Austria

^2^Division of Cardiology, Department of Medicine II, Medical University of Vienna, Vienna, Austria

^3^Division of Endocrinology and Metabolism, Department of Medicine III, Medical University of Vienna, Vienna, Austria

^4^Department of Medicine, Landesklinikum Scheibbs, Scheibbs, Austria

**Supplementary Table 1** Primary and secondary study outcomes in patients with normoglycemia, prediabetes and diabetes based on baseline fasting glucose levels (normoglycemia: ≤5.5mmol/L or ≤99mg/dL, prediabetes: 5.6-6.9 mmol/L or 100-125mg/dL and diabetes: ≥7.0 mmol/L or ≥126mg/dL).

| Study Endpoints | Normoglycemia  (n=569) | Prediabetes  (n=440) | Diabetes  (n=2152) | Pre-diabetes vs Normoglycemia | | Diabetes vs Normoglycemia | |
| --- | --- | --- | --- | --- | --- | --- | --- |
|  |  |  |  | unadjusted HR | adjusted HR | unadjusted HR | adjusted HR |
| All-cause mortality | 32 (5.6) | 28 (6.4) | 224 (10.4) | 1.1  0.66-1.83) | 1.02  (0.61-1.72) | 1.94  (1.34-2.81) | 1.62  (1.10-2.39) |
| All-cause mortality or myocardial infarction | 55 (9.7) | 53 (12.0) | 381 (17.7) | 1.07  (0.75-1.52) | 1.00  (0.69-1.43) | 1.78  (1.37-2.30) | 1.51  (1.15-1.98) |
| Cardiovascular death, myocardial infarction, unstable angina, heart failure, or resuscitated cardiac arrest | 61 (10.7) | 60 (13.6) | 427 (19.8) | 1.26  (0.88-1.8) | 1.17  (0.81-1.68) | 1.98  (1.51-2.58) | 1.63  (1.23-2.16) |
| Cardiovascular death, myocardial infarction, stroke | 56 (9.8) | 61 (13.9) | 419 (19.5) | 1.40  (0.97-2.01) | 1.32  (0.91-1.91) | 2.12  (1.602  -2.80) | 1.83  (1.36-2.45) |
| Cardiovascular death | 20 (3.5) | 25 (5.7) | 181 (8.4) | 1.58  (0.88-2.84) | 1.49  (0.82-2.73) | 2.50  (1.57-3.96) | 2.07  (1.28-3.35) |
| Myocardial Infarction | 43 (7.6) | 35 (8.0) | 257 (11.9) | 1.04  (0.67-1.63) | 0.97  (0.61-1.54) | 1.68  (1.21-2.32) | 1.44  (1.0-2.03) |
| Heart failure | 6 (1.1) | 7 (1.6) | 57 (2.6) | 1.47  (0.50-4.38) | 1.55  (0.49-4.90) | 2.64  (1.14-6.13) | 1.81  (0.71-4.62) |
| Stroke | 3 (0.5) | 10 (2.3) | 55 (2.6) | 4.25  (1.17-15.45) | 4.0  (1.09-14.51) | 5.07  (1.59-16.20) | 4.47  (1.38-14.52) |
| Initiation of new dialysis | 3 (0.6) | 3 (0.7) | 54 (2.7) | 1.28  (0.26-6.36) | 1.70  (0.32-8.9) | 5.12  (1.6-16.37) | 2.54  (0.75-8.55) |

**Supplementary Figure 1** Association between glycated hemoglobin (%) at baseline and risk of all-cause death.

**Supplementary Figure 2** Cumulative incidence of patients progressing from normoglycemia or prediabetes to diabetes, according to HbA1c levels at follow-up visits.

**Supplementary Figure 3** All-cause mortality between patients with prediabetes with or without progression to diabetes within the observation period.ss
